# Object-Gaze Distance: Quantifying Near- Peripheral Gaze Behavior in Real-World Applications

**DOI:** 10.16910/jemr.14.1.5

**Published:** 2021-05-19

**Authors:** Felix S. Wang, Julian Wolf, Mazda Farshad, Mirko Meboldt, Quentin Lohmeyer

**Affiliations:** ETH Zurich, Switzerland; Balgrist University Hospital, Switzerland; University of Zurich, Switzerland

**Keywords:** mobile eye tracking, peripheral vision, areas of interest, machine learning, object detection, visual expertise

## Abstract

Eye tracking (ET) has shown to reveal the wearer’s cognitive processes using the measurement of the central point of foveal vision. However, traditional ET evaluation methods have not been able to take into account the wearers’ use of the peripheral field of vision. We propose an algorithmic enhancement to a state-of-the-art ET analysis method, the Object- Gaze Distance (OGD), which additionally allows the quantification of near-peripheral gaze behavior in complex real-world environments. The algorithm uses machine learning for area of interest (AOI) detection and computes the minimal 2D Euclidean pixel distance to the gaze point, creating a continuous gaze-based time-series. Based on an evaluation of two AOIs in a real surgical procedure, the results show that a considerable increase of interpretable fixation data from 23.8 % to 78.3 % of AOI screw and from 4.5 % to 67.2 % of AOI screwdriver was achieved, when incorporating the near-peripheral field of vision. Additionally, the evaluation of a multi-OGD time series representation has shown the potential to reveal novel gaze patterns, which may provide a more accurate depiction of human gaze behavior in multi-object environments.

## Introduction

Eye tracking (ET) has proven to be a powerful tool for analyzing behavioral patterns, both in laboratory and in real-world environments (3, 5). It has demonstrated the ability to reveal and quantify cognitive strategies during goal-oriented tasks (18). Due to easier accessibility of the technology in recent years, it has been increasingly used for the investigation of visual expertise in medicine (4, 9), in order to increase the effectiveness and diagnostic accuracy of physicians-in-training (33).

One of the most established ET methods is the Area of Interest (AOI), sometimes referred to as regions of interest, analysis, where the gaze point is mapped to predefined areas that are of interest to the evaluator (16). How AOIs are defined and the way in which the data is subsequently used to draw conclusions, varies from case to case (2). For manual tasks, AOIs can be hand-held objects, interface buttons, or screens - generally, any visible parts of the physical environment. By mapping AOI Hits, where each fixation is assigned to the looked-at AOI, the gaze data is given semantic meaning (26). The resulting fixation count can be subsequently used to calculate a variety of AOI metrics, dwell times (1) or AOI revisits (12), which in turn enable more in-depth behavioral analysis.

Even though it has been shown that individuals can perceive targets without looking at them directly (10), current gaze matching methods are restricted to the use of a mere fraction of the human eyes' visual field, neglecting the use of peripheral vision. Starting from the center of the gaze, the human fields of vision consist of: foveal vision (< 2° diameter), colloquially called sharp vision, and peripheral vision (> 2°), including parafoveal (~ 5°-9°) and perifoveal (~ 9°-17°) vision (31, 34).

For mobile or wearable ET systems, AOI Hit analysis has always been more challenging due to the relative movements between the head and the AOIs (e.g. hand-held objects) in the recorded scene. The mapping of the gaze point to AOIs is therefore predominantly carried out manually, making the measurement of the use of peripheral vision unfeasible. During manual gaze mapping, an analyst’s decision is limited to whether the gaze point, representing the foveal vision, lies within the constraints of the pre-defined AOIs, or not. Contrary to findings from previous studies, this evaluation method limits our attentional capacities to register one object at a time.

Recent applications of deep convolutional neural networks have used image segmentation to detect objects in mobile ET recordings and provided a way to automate the mapping of the gaze onto these detected AOIs (35). The automated gaze-object mapping algorithm processes each fixation as a pair of x- and y coordinates. Until now, this method has reduced foveal vision to a single pixel within the recorded scene, which has been shown to lead to an omission of a majority of fixations, even though minimum calibration requirements were fulfilled (11).

The noise-robustness of eye tracking systems (17) and the choice of the size and shape of AOIs (15) for optimal AOI mapping has long preoccupied researchers. During gaze mapping, AOIs that are drawn closely around objects of interest can cause false negatives (fixations that belongs to the object but is not mapped), while objects with large AOI paddings can equally cause false positives (fixations that belong to other objects) (24). Researchers have explored various ways to optimize AOI sizes to increase noise-robustness during AOI mapping, most popularly by increasing the area around objects by specific margins (15). However, to our knowledge, no known method exists that enhances data mapping by measuring the distance of the gaze point to AOIs and the object’s position within an operator’s field of vision.

In the case of multi-object environments, an operator can manipulate several small tangible objects simultaneously. Consequently, experienced operators have been shown to expand their visual field to incorporate areas using their peripheral vision (20). As a result, the gaze point can often lie between several of these objects without fixating on a specific one. Using the binary matching of a one-fixation-to-one-AOI approach, as traditional methods do, the central foveal attention to single objects can be mapped, while neither the information of the attention nor the position of other objects that lie within the close vicinity of the gaze point can be recorded. It is therefore unknown whether, in these environments, and without the consideration of the near-peripheral field of vision, is sufficient to represent the operator's actual gaze behavior. To include the fields of near-peripheral vision in the data evaluation and to make AOI analysis applicable in multi-object environments, we hereby propose a new gaze metric: the Object-Gaze Distance (OGD). The OGD creates a positional relationship between the gaze point and the AOIs, by calculating a 2D Euclidean pixel distance between the gaze point and the segmented mask of each object of interest, provided through an image segmentation algorithm. This shall allow us to evaluate the position of an object within the operator’s field of vision, while enabling the spatial mapping of all fixations to each AOI, increasing the amount of information that we can leverage for human behavior analysis.

In this article, we compared the proposed method to a state-of-the-art automated AOI Hit method and quantitative assessments on the number of fixation after semantic mapping are made, using mobile eye tracking recordings from a real surgical case as an exemplary case. Furthermore, we conducted a qualitative investigation on novel OGD based behavioral patterns to evaluate the operators’ gaze behavior in the given task. The goal was to examine and discuss the potential benefits and limitations of the proposed gaze metric with respect to the use of peripheral vision in multi-object environments and its suitability for wearable support systems.

## Methods

In this section, the details of how the data was recorded in the surgical environment, the functionality of the presented algorithm, and the analysis performed for the evaluation of the OGD metric, are described.

### Participants

Eye movements from two experienced spine surgeons (male, aged 37 and 39, respectively), from a (withheld for blind review) hospital, were recorded during spondylosis on real patients. Each surgeon had participated in more than 1200 spondylosis procedures prior to data recording.

### Stimulus

**Figure 1.**
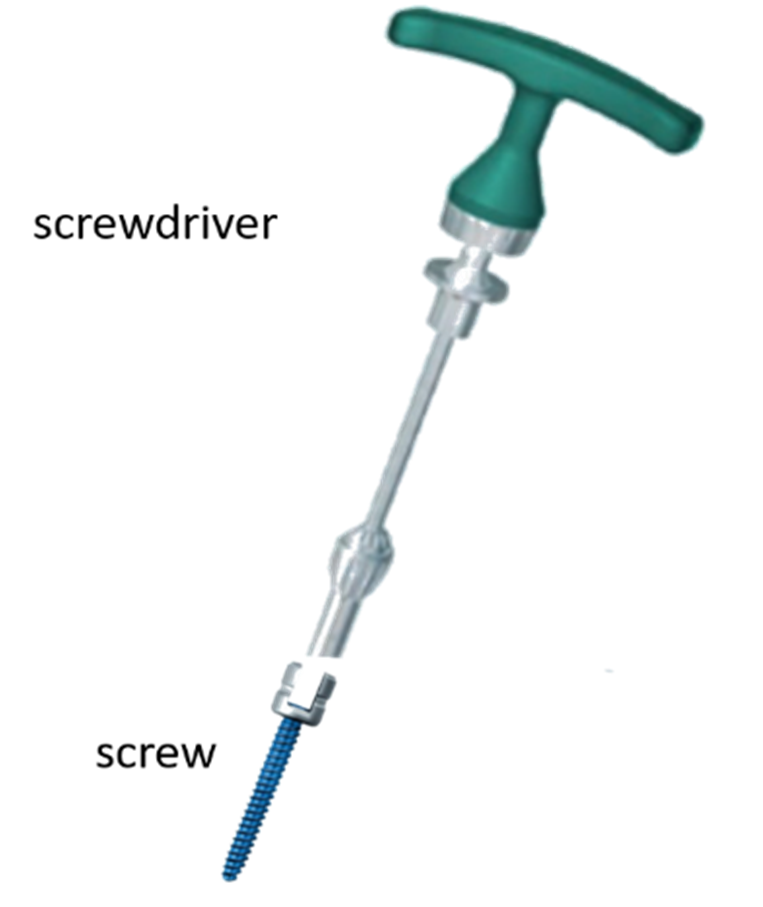
The main surgical tools used during the spondylosis screw placement. A specialized operational screwdriver (DePuy Synthes) is connected to the medical screw during placement and disconnected after the successful placement

Spondylosis is a common surgical intervention for spinal stabilization in patients with debilitating back pain and neurogenic symptoms. In this procedure, medical screws are commonly placed in at least two vertebrae and connected by a rod to decompress the neuronal structures. In this article, each evaluated procedure of the data set includes the placement of one screw using a specialized screwdriver. Data recording started from the moment the surgeon first grabbed the screwdriver and ended when the placement of the screw was concluded by the removal of the tool. Figure 1 shows a medical screw and screwdriver used during spinal screw placement, which was defined as the two primary AOIs used for the subsequent investigations, in the procedural context. As the surgeons’ visual attention is mostly located within the wound during surgical execution, we expect that choosing the open wound as an additional AOI provides only limited informational value, similar to the background. Splitting the wound into several sub-AOIs would allow a more fine-grained AOI Hit analysis, but cannot be done reliably and comparably due to the complexity of the surgical scene and was therefore omitted.

### Eye Tracking System

Data was collected using SMI’s ETG 2 eye tracking glasses with a frontal camera sampling rate of 60 Hz and a scene resolution of 1280 x 960 px (viewing angle: 60° horizontal, 46° vertical). Gaze point measurement accuracy is 0.5° over all distances.

During data recording, ET glasses were tightened by the moderator, using the device’s headband to prevent device slippage. We conducted the calibration using the SMI recording unit and a three-point calibration, where the wearer was asked to fixate three specific markers (top-left corner, top-right corner, and the middle of the bottom edge). During each marker fixation, the experimenter manually confirmed these marker locations on a live-view of the scene camera on the recording device. Afterward, the experimenter made sure that both eyes were clearly visible on the eye camera recordings and calibration was validated using a three-point validation of specific points within the task environment. If calibration accuracy was not sufficient, calibration and validation were repeated.

The eye tracking accuracy of all procedures was 96.68 ± 0.93 %. However, even though tracking accuracy was sufficiently high, the lighting conditions in the operating room environment proved to be challenging for the eye tracking equipment. Due to the placement of the bright operation lights above the surgeons, we observed that incoming IR light occasionally caused a gaze point offset. To counter this problem, an infrared shield was attached to the top of the ET glasses halfway through data recording, which successfully increased gaze point accuracy. Consequently, in order to allow the most accurate gaze analysis, we only included those trials that showed no gaze shifts in the recordings. Thus, for the final analysis 11 of 18 recordings were used, while 7 recordings had to be excluded.

### Computation of the Object-Gaze Distance (OGD)

**Figure 2.**
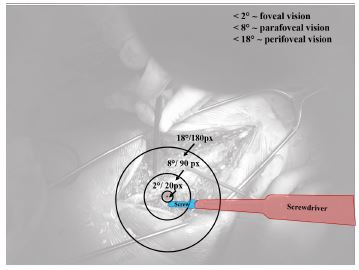
Stimuli of the surgical procedure, showing the task related AOIs screw (blue) and screwdriver (red) and a visualization of the foveal, parafoveal and perifoveal field of vision around the gaze point (red dot).

The semantic mapping of fixations for the AOI Hit method was implemented using the automated AOI mapping algorithm cGOM (35). The algorithm detects and segments pre-trained objects, here the screw and the screwdriver, using the Mask R-CNN network (14) and, subsequently, determines whether the gaze of each fixation lies within the constraints of the 2D pixel (px) coordinate matrix of the segmented object masks. If gaze coordinates do not coincide with any AOI matrices, fixations are assigned the label ‘background’ and omitted from further gaze behavior analysis. Figure 2 shows the AOIs *screw* (highlighted in blue) and *screwdriver* (highlighted in red), and the gaze point (red dot) as detected by the cGOM algorithm within the scenery.

For each fixation, the OGD extends AOI mapping by calculating a positional relationship between the gaze coordinate and an AOI in the image plane. The distance is expressed through the minimal 2D Euclidean px value between each AOI mask and the gaze coordinate of a fixation. Thus, in contrast to the traditional AOI Hit mapping method, all fixations are now mapped to each pre-trained object of interest, continuously throughout the whole trial. The value of each OGD can range between 0 px, representing an AOI Hit, and 1600 px, which equals the length of the diagonal of the SMI ET camera resolution. In the case that one object was not detected by the algorithm in the video frame at all, the OGD was set to take on the value of 1600 px, which is the upper threshold for the subsequent analysis.

The training of the convolutional neural network started with the initial weights of the already pre-trained MS COCO data set (21). This so-called transfer learning approach has been the state-of-the-art in image segmentation in cases with limited data sizes (13). Pre-trained CNNs have been shown to outperform fully trained networks in image classification (29) because it allows researchers to make use of the extracted features that have already been learned on a large data set (8), while building the classification layer in a more accurate timesaving way (27). For our surgical case, we used 420 images of the screw insertion, 82 % of which were used for training and 18 % for the validation of the algorithm. We selected these images from screw placement recordings using a random frame extraction algorithm and revised them to assure that each distinct scene of the process was represented in the image data set. After training was completed, the new weights were used to evaluate the target data set. The target data set consisted of 11 spinal screw placement procedures and a total of 290 fixations (average fixation duration = 0.65 ± 0.86 s). To assess the quality of the object segmentation 100 frames were randomly selected, labeled manually with masks, and, finally, compared with the predictions. The evaluation of the mask quality was based on the Intersection over Union (IoU) metric, which is calculated by dividing the area of overlap between the predicted and the manually labeled mask by their area of union. The IoU was calculated based on 100 ground truth images.

### The Peripheral Field of Vision

For the different fields of vision the following terminology is used in the subsequent analysis: Foveal vision (< 2 °) and peripheral vision, including parafoveal (< 9 °) and perifoveal (< 18°) vision (31, 34). Figure 2 shows a visualization of the different fields of vision, each displayed as a ring around the gaze point. With the given ratio between viewing angle and scene resolution of the ET glasses, we transformed the fields of peripheral vision into the px ranges shown in Table 1. Here, the near-peripheral vision is restricted to a 60° degrees visual angle, which was given by the maximum measurable viewing angle of the SMI ET glasses.

**Table 1.** Calculated thresholds for different fields of vision in pixel, based on the resolution of the recording device.

*Fields of vision*	*Degree of Diameter [°]*	*Pixel Threshold OGD [px]*
Foveal	< 2	~20
Parafoveal	< 9	~90
Perifoveal	< 18	~180
Near-Peripheral	< 60	~640

### Data Analysis

In this article, the benefits of OGD as an improved extension of traditional AOI Hit mapping analysis are evaluated in three parts. First, the computed AOI Hits and OGDs for AOIs screw and screwdriver are visualized for two exemplary screw placements, in order to evaluate the number of mapped fixations in each method. Second, a parameter study was conducted to investigate the number of fixations that can be exploited for behavioral analysis, for both the AOI Hit and OGD methods. We calculate the fixation rate (FR) using AOI Hits and the rate of fixations found within the foveal and near-peripheral field of vision, to compare the number of interpretable fixations and to thereby infer the gained informational value. The FR is calculated using Equation 1 (30).

(1) $$FR=\;\frac{\text{Total Number of Fixations assigned to AOI}}{\text{Total Number of Fixations}}$$

The px distance parameter was varied between 0 and 1600 px, using 10 px steps. Different fields of vision, such as foveal, parafoveal and perifoveal vision were utilized as points of reference in the analysis. For reasons of readability, only results between 0 and 200 px, which corresponds to the perifoveal field of vision, are discussed in this article. Third, we explore the benefits of a multi-object Object-Gaze Distance (multi-OGD) visualization for AOIs screw and screwdriver by qualitatively examining possible novel gaze patterns. Characteristic trends of the multi-OGD curves – for example, the simultaneous increase of gaze object distances – are shown and discussed in the given data set.

## Results

**Figure 3.**
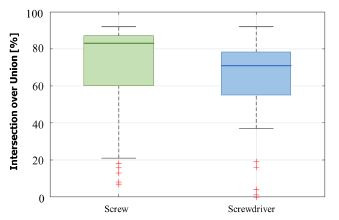
Boxplot showing the distribution of the calculated intersection over union (IoU) of the segmented masks for AOIs screw and screwdriver.

Figure 3 shows the distribution of the calculated IoUs of AOI *screw (70.65 ± 23.91 %)* and AOI *screwdriver (62.93 ± 23.48 %)*.

### Comparison of AOI Hits and Object-Gaze Distance

**Figure 4.**
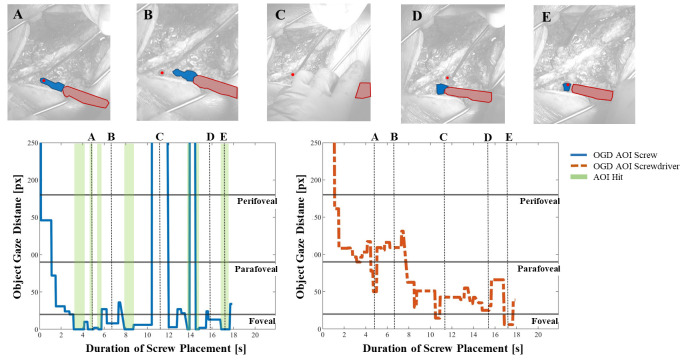
The Object-Gaze Distance (OGD) over the course of one exemplary procedure in pixels (px), for AOIs screw (blue solid line) and screwdriver (red dotted line). AOI Hits (OGD = 0 px) are highlighted using green bars and show that no AOI Hits were detected for screwdriver. A, B, C, D and E show snapshots from the recordings with the segmented AOI masks and the gaze point (vertical dotted lines). Horizontal lines indicate the threshold pixel distance for foveal (< 20px), parafoveal (< 90px) and perifoveal vision (< 180px).

Figure 4 shows the OGDs of AOIs *screw* and *screwdriver* during one screw placement procedure in blue and red, respectively. Fixations that were mapped as AOI Hits by the cGOM algorithm are marked as green bars and the near-peripheral fields of vision are shown as horizontal lines. Additionally, five scene images from the procedure recordings are provided with dotted vertical lines, where the gaze point is visualized by a red dot and detected AOIs *screw* with a blue and *screwdriver* with a red mask.

The OGD of AOI *screw* shows that the screw was within a close distance, mostly within the foveal field of vision, throughout the majority of the procedure. In comparison, the AOI Hit bars (i.e. OGD = 0 px) show that the cGOM algorithm has detected only 7 fixations, two of which are visualized in images A and E. The distance between the AOI *screw* mask and gaze coordinate in images B and D are shown to be still within the foveal field. However, since these fixations were not intersecting the AOI mask area, no AOI Hit could be mapped. In image C, AOI *screw* was not detected by the neural network within the image, leading to a distance value outside of the frame of reference (1600 px). The progression of the OGD curve of AOI *screwdriver* indicates that the tool was further away from the gaze point, mostly within the perifoveal and parafoveal field of vision. Moreover, no direct AOI Hits were detected by the automated mapping tool on AOI *screwdriver* throughout the entire screw placement procedure. The distance between AOI *screwdriver* and the gaze point can be seen to decrease continuously as the screw placement progresses.

**Figure 5.**
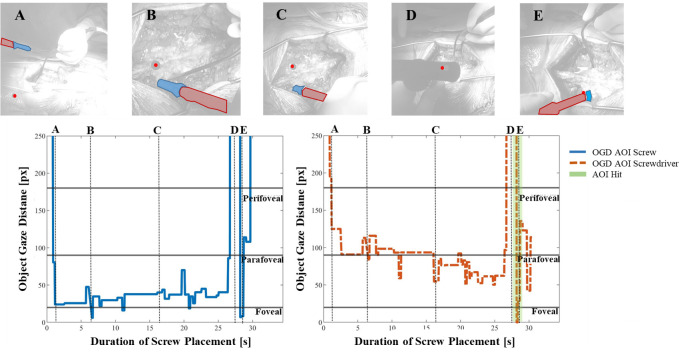
The OGD of a second exemplary spondylosis procedure, showing that only one overall fixation was detected as an AOI Hit (OGD = 0 px) for AOI screwdriver, while no AOI Hits could be detected for AOI screw. Again, the OGD for AOIs screw (blue solid line) and screwdriver (red dotted line) are provided and A, B, C, D and E show snapshots from the recordings with the segmented AOI masks and the gaze point (vertical dotted lines).

Figure 5 shows the OGDs of AOIs *screw* and *screwdriver* for the second exemplary screw placement procedure. Compared to the previous case, no AOI Hits were detected for AOI *screw.* The AOI was found within the parafoveal field of vision for the majority of the fixations. Images B and E show AOI *screw* inside the foveal field of view, while in images A and C it is located further away. In image D, the screw could not be detected by the neural network, which is reflected by a distance value of 1600 px.

For AOI *screwdriver,* cGOM was able to map a single fixation as an AOI Hit, towards the end of the procedure (image E). The progression of the OGD curve closely resembles that of the first screw placement, where the distance between *screwdriver* and gaze indicates the tool placement within the near-peripheral field of view. The distance reduced gradually with increasing trial duration. For images A, B, and C, the *screwdriver* was located further away than the *screw*, which is explained by the fixed relative position between the entry wound, the screw, and the screwdriver. Similar to AOI *screw*, the screwdriver object could not be detected in image D, leading to no AOI mask and a distance value of 1600 px.

### Consideration of Fixations inside the Near-Peripheral Fields of Vision

**Figure 6.**
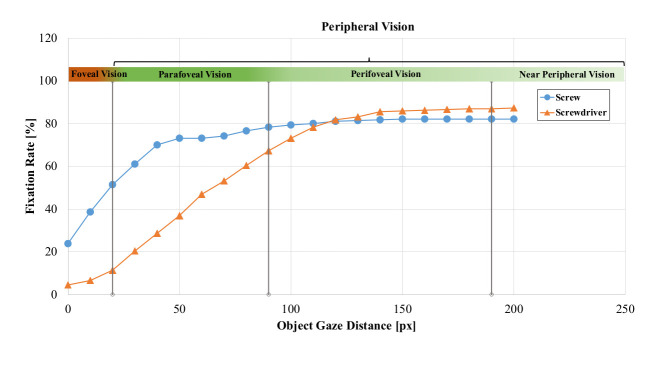
Fixation rate (FR) of the OGD for AOIs screw and screwdriver, averaged over the whole data set in 10 pixel (px) steps. Vertical lines indicate the threshold distances for foveal (< 20px), parafoveal (< 90px) and perifoveal vision (< 180px). A colored bar over the curve indicates different fields of vision.

From the 11 analyzed procedures of the data set, 290 fixations were registered with a recording time of 243.63 s (average per procedure: 22.15 ± 6.79) for all procedures. Figure 6 shows the *fixation rate* (*FR)* in 10 px steps, varying from 0 to 200 px and averaged over all trials. At distance 0 px, the equivalent to the *FR* of AOI Hits, the *FR* of AOI *screw* was 23.8 %, while the *FR* of AOI *screwdriver* was 4.5 %, leaving more than 70 % of unaccounted fixations. For fixations within a range of distance 0 < 20 px, the approximate field of foveal vision, the *FR* of mapped fixations increased to 51.4 % and 11.4 %, respectively. Consequently, for half of all fixations, AOI *screw* were found inside the surgeon’s main area of sharp vision and thus, in the area of visual attention. Moreover, even though the surgical screwdriver was the surgeon’s main tool of manual manipulation, for only a small number of fixations, the AOI *screwdriver* was found within the area of foveal vision. For distances within the range of 0 < 90 px, the parafoveal field of vision, the *FR* of mapped fixations increased to 78.3 % and 67.2 %, respectively.

Expanding the analysis to inside the perifoveal field of vision (0 < 180 px), the *FR* of AOI *screw* was 82.1 % and the *FR* of AOI *screwdriver* was 86.9 %.

### Categorization of Novel Visual Patterns for Multi-Object Environments

**Figure 7.**
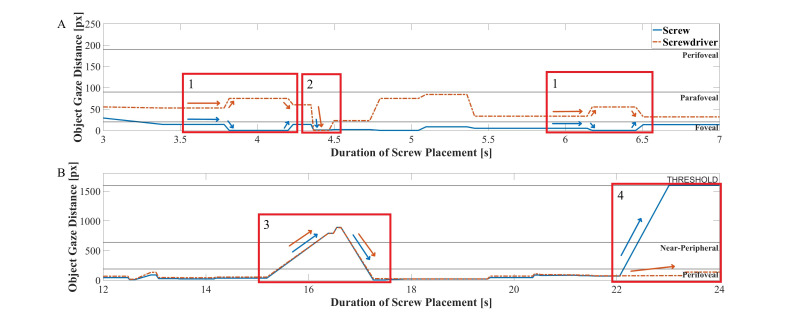
Multi-Object-Gaze Distance (multi-OGD) graph for simultaneous analysis of OGDs of AOIs screw and screwdriver. Arrows in the color of the corresponding Area-of-Interest indicate the characteristic direction of movement for each object. Panel A shows two examples of gaze pattern 1), a characteristic movement of the OGDs of screw and screwdriver in opposite directions and one example of gaze pattern 2, a simultaneous decrease of both OGDs, including the AOI Hit (0 px) on one of the AOIs. Panel B shows one example of pattern 3, simultaneous increase and decrease of both OGDs outside of the near-peripheral field and one example of pattern 4, where the OGD of only one AOI increases to outside of the field of view, while the other stays close to the gaze point.

A qualitative analysis of the operators’ gaze behavior using the multi-OGD was conducted, which combines the distance of the gaze to both AOIs into a single figure. We discovered that recurring trends might exist for multi-OGDs inside the perifoveal field of vision. Figure 7 presents an excerpt of the multi-OGD for one spinal screw placement. The qualitative analysis of the graphs yielded two gaze patterns in both the near-peripheral and the peripheral field of vision. Gaze patterns in the near-peripheral field of vision are shown in Figure 7A:

1. The two task relevant objects have a different relative positions to the gaze point, AOI *screw* being closer and AOI *screwdriver* further away. An AOI Hit on AOI *screw* is made, moving the distance to 0 px, while the distance to AOI *screwdriver* increases. The gaze then reverts back to a similar position as before the AOI Hit. The distance curve behavior resembles a quick glance onto one object, possibly for status checking purposes. The pattern is shown twice in Figure 7 and was found 16 times (mean: 1.45 ± 0.89) over all procedures.
2. The gaze distance to both AOIs decreases simultaneously, until both lie within the foveal field of vision, with an AOI Hit on one AOI, *screwdriver*. The gaze point has moved to the point of interconnection between these two objects. The pattern was found an overall of 16 times (mean: 1.09 ± 1.38) over all procedures.

Gaze patterns in the peripheral field of vision are shown in Figure 7B:

3. The gaze point distance to both objects increases simultaneously from the inside the perifoveal field of vision into the peripheral field of vision. After a few fixations the OGDs decrease back to within the perifoveal field of view, showing an equal distance to both AOI *screw* and *screwdriver*. This gaze pattern was found 3 times (mean: 0.27 ± 0.45) in all procedures.
4. Both objects are located close to the gaze, within the perifoveal field of vision. The OGD of AOI *screw* increases to a value beyond the defined pixel threshold, while the OGD of the object screwdriver remains within the same relative distance. This trend shows the disappearance of one object from the operator’s field of view. In Figure 7B, this pattern occurs at the end of the trial, potentially indicating the end of the procedure, when the screw is driven into the spine and concealed by the surrounding flesh. This gaze pattern was found in all but one of the 11 procedures (mean: 1.00 ± 0.43).

## Discussion

The goal of this article was to investigate the benefits of the newly introduced eye tracking data mapping method *Object-Gaze Distance.* Possible enhancements over traditional AOI mapping methods were explored and the suitability for an in-depth understanding of human behavior in multi-object environments was assessed.

Our results have shown that in cases where the majority of fixations cannot be mapped as an AOI Hit onto any object of interest used by the surgeon, an alternative method is required to acquire sufficient gaze data. In our case, choosing the wound area as an additional AOI could account for most of the fixations that would otherwise be mapped to AOI *background*. Contrary to the OGDs, the newly achieved high number of mapped fixations does not provide additional informative value about the surgeons’ gaze behavior within the wound.

When working with AOI methods, noise robustness and choice of AOI size for optimal mapping is an important topic. In their work, Hessels et al. (15) have shown that by increasing the area around objects, using padding of 1.5 degrees, more fixations can be mapped to the chosen AOIs. Thus, for similar types of stimuli, they concluded that, by adopting larger AOIs, the most objective noise-robust results could be achieved. While in remote eye tracking studies of sparse static stimuli, the degree, size, and positioning of AOIs can be effortlessly adjusted by the analysts, in mobile eye tracking object shapes are highly dynamic and objects of interest can be small and closely positioned to one another. Therefore, increasing the AOI size around objects of interest can result in areas that exceed the size of the actual objects by several orders of magnitude. In our study, we assume that the most accurate depiction of an operator’s gaze behavior can only be achieved using a close contour AOI production method, such as Mask R-CNN and cGOM (35), that continuously adjusts AOI sizes to the actual object size within the dynamic scene. One advantage of our presented method is that it can be equally used for the analysis of AOI Hits, if traditional metrics are of interest to the researchers. Here, we would propose that the gaze point coordinate is extended to include the area of foveal vision using an OGD < 20 px, or an OGD = 0 px with additional AOI padding.

If multiple objects are close to one another, as were *screw* and *screwdriver* in our case, AOI overlaps can occur, leaving the analysts to predefine which of these AOIs the fixations will be assigned to (24). In remote eye tracking, multiple options have been suggested to deal with these overlapping AOIs. Holmqvist et al. (16) advise including a free space of 1.5 degrees size between AOIs to decrease the number of false-positive fixation assignments. Clarke et al. (6) have dealt with the overlap problem by assigning these fixations to the smallest AOI containing it, while Yun et al. (36) have allowed these fixations to be assigned to more than one AOI. Here, our OGD method removes the necessity of predefining an assignment logic, by enabling a simultaneous mapping of fixations to multiple AOIs. We are therefore able to gain the information of each object’s position within the wearer’s field of vision while evading the AOI overlap problem.

As each AOI evaluation relies heavily on the accuracy of the eye tracker, even small offsets and device slippage (23) can lead to a loss of data. While the binary assignment of each fixation to only one-AOI-at-a-time can limit the amount of information that can be gained from the operator’s visual behavior, the OGD can produce a substantial amount of processable (note that this is not equal to accurate) data even in the case of accuracy losses. Our proposed method, therefore, provides an enhancement to the current state-of-the-art, while it should by no means serve as a replacement to the acquisition of high-quality data. In the future, data accuracy losses could be simulated by adding Gaussian noise to the acquired data, to assess the robustness of OGDs and the resulting time series in these circumstances (15).

We want to mention here that the OGD does not per se increase the collected data (the number of fixations will not change), but in essence, multiplies the amount of data that can be used for further behavioral analysis, creating time-series data for each pre-trained object. In future investigations, these time-series shall be analyzed using more data to verify the current findings and to automate behavior recognition. Mussgnug, Singer, Lohmeyer and Meboldt (22) have shown in their work that a calculated 2D distance between gaze and hands, using an RGB approach, can be utilized for the automated detection of cognitive demanding phases. Using time-series classifiers (TSC), such as Dynamic Time Warping (7, 32), support vector machines (25), or image encoding based classifiers (19), algorithms could be trained for the detection of behavioral patterns in human tasks, using these gaze distances as an input parameter.

In conclusion, we showed that the existing state-of-the-art AOI evaluation method can reach its limits when used in a challenging real-world multi-object application. Our results imply that through the use of close contour object masks and the subsequent introduction of a measurable distance between gaze point and objects, the extend of a surgeon’s use of his foveal and peripheral field of vision can be visualized more accurately. Consequently, the information on the location of an object within the surgeon’s near-periphery can now be automatically quantified for an adequate behavioral analysis in multi-object environments.

In the analyzed surgical procedure, the analysis of multi-OGDs indicated novel gaze patterns, which have allowed deeper insights into the operator’s behavior during tool handling. Even though the implications from the analysis resulted from a limited sample size of medical experts, the automated measurement of the object gaze distance nonetheless provides a novelty in the field of expert visual behavior analysis. Previously, several studies comparing experts and novices have shown that experts make more use of their near-peripheral vision as their experience grows, registering information from AOIs they are not directly looking at (20, 28). Consequently, for expertise research, the OGD algorithm enhances state-of-the-art behavioral analysis by enabling continuous automated measurement of experts’ use of peripheral vision.

In the near future, it is expected that technological advancements in processing speeds and cloud computing will make it possible for wearable devices to incorporate the automated evaluation of these behavioral patterns. This will forge the way for reliable real-time step detecting support systems for more effective training and analysis of physicians-in-training, increasing the support in complex multi-object environments.

### Limitations

Due to limited availability of spondylosis expert surgeons, along with often challenging lighting conditions, the analysis contains the data of only two experts. The analysis of more expert eye movements could help to confirm the quantitative findings of the multi-OGD. Furthermore, the object detection algorithm might be susceptible to fluctuations of segmented object masks due to the size of our used training set. While this creates a concern to a certain extent, we firmly believe that additional time invested into image labeling, as well as advances in the neural network training methodologies, will further increase algorithm accuracy. The authors are aware that the OGDs presented in this article are based on a 2D calculation of the pixel distance between object mask and gaze point, which can lead to a parallax in the operator’s actual point of attention. With modern wearable systems available on the market, such as the Microsoft HoloLens 2, which include depth perception, two-dimensional inaccuracies will soon be accounted for.

### Conclusion

Our introduction of the object-gaze distance has shown to substantially enhance conventional AOI evaluation in a real-world application in several ways. Quantification of near-peripheral gaze behavior not only allowed for more robust and in-depth analysis of gaze data, but also the increase of obtained data quantity by several factors. Simultaneous representation of multi-object distances has led to the discovery of possible novel recurring gaze behaviors. Thus, we are convinced that the presented method for the measurement of near-peripheral vision can significantly improve the application of mobile ET in complex, cognitively demanding scenarios and allow a more accurate depiction of operators’ visual behavior, such as during medical procedures.

### Ethics and Conflict of Interest

Ethics have been approved by the ethics committee Zurich (BASEC No. Req-.2018-00533). The authors declare that they have no conflict of interest.

### Acknowledgements

This work is part of the SURGENT project and was funded by University Medicine Zurich/ Hochschulmedizin Zürich. We would like to thank Diana Schluep for her involvement in the collection of the eye tracking data.
